# Clinical Significance of Distant Metastasis-Free Survival (DMFS) in Melanoma: A Narrative Review from Adjuvant Clinical Trials

**DOI:** 10.3390/jcm10235475

**Published:** 2021-11-23

**Authors:** Simone Amabile, Gabriele Roccuzzo, Valentina Pala, Luca Tonella, Marco Rubatto, Martina Merli, Paolo Fava, Simone Ribero, Maria Teresa Fierro, Paola Queirolo, Pietro Quaglino

**Affiliations:** 1 Section of Dermatology, Department of Medical Sciences, University of Turin, 10126 Torino, Italy; simone.amabile@edu.unito.it (S.A.); v.pala@unito.it (V.P.); luca.tonella@unito.it (L.T.); rubattomarco@gmail.com (M.R.); merlimartina93@gmail.com (M.M.); fava_paolo@yahoo.it (P.F.); simone.ribero@unito.it (S.R.); mariateresa.fierro@unito.it (M.T.F.); pietro.quaglino@unito.it (P.Q.); 2 Division of Medical Oncology for Melanoma, Sarcoma, and Rare Tumors, European Institute of Oncology (IEO), European Istituto di Ricovero e Cura a Carattere Scientifico (IRCCS), 20141 Milan, Italy; paola.queirolo@ieo.it

**Keywords:** melanoma, DMFS, metastatic melanoma, adjuvant, distant metastasis-free survival, relapse-free survival

## Abstract

Cutaneous melanoma is the most dangerous skin cancer, with high death rates in advanced stages. To assess the impact of each treatment on patient outcomes, most studies use relapse-free survival (RFS) as a primary endpoint and distant metastasis-free survival (DMFS) as a secondary endpoint. The aim of this narrative review of the main adjuvant studies for resected stage III/IV melanoma, with a specific focus on DMFS, is to evaluate DMFS trends and their potential association with RFS, identify which treatments are possibly associated with better outcomes in terms of DMFS and their potential predictive factors, and discuss DMFS trends in terms of patient management in daily practice. We outline the impact of each available treatment option on DMFS and RFS according to the years of follow-up and compare data from different studies. Overall, the trends of DMFS closely follow those of RFS, with most patients relapsing at visceral rather than regional sites. As it captures the burden of patients who develop distant relapse, DMFS could be considered a primary endpoint, in addition to RFS, in adjuvant trials, identifying patients whose relapse is associated with a worse prognosis and who may need further systemic treatment.

## 1. Introduction

In the last decades, the incidence of cutaneous melanoma (CM) has gradually increased, with 324,635 new cases and 57,043 deaths in 2020 [1,2]. Currently, primary prevention and early diagnosis remain the main defenses to improve the prognosis of melanoma patients. Great efforts are aimed at identifying the best predictive and prognostic indices, such as the latest American Joint Committee on Cancer (AJCC) Melanoma Staging [3,4]. In recent years, research on adjuvant and neoadjuvant melanoma treatment has made much progress, but the major issue is the translation of innovations from trials into clinical practice. Patients eligible for adjuvant therapy in real-life settings differ substantially from patients originally included in clinical trials, as their stage is now based on the AJCC 8th classification instead of the AJCC 7th edition, and most of them would not have undergone complete lymph node dissection (CLND) [5].

Melanoma treatment is based on the tumor stage, which is defined according to the TNM classification described in the 8th edition of AJCC, following excisional biopsy of the suspicious lesion and histological and instrumental examinations.

Patients with pT ≥ 1b or pT1a with regression > 75% undergo sentinel lymph node biopsy. In the past years, patients who were positive for melanoma cells could undergo CLND, but currently, following the Multicenter Selective Lymphadenectomy Trial II (MSLTII) and the Dermatologic Cooperative Oncology Group trial (DECOG), CLND is no longer carried out [6].

Despite early diagnosis, the survival of melanoma patients remains 88%, 82% and 75% for stages IIA, B and C and 88%, 77%, 60% and 24% for stages IIIA, B, C and D, respectively, even after radical interventions, such as excision or lymphadenectomy [7]. These data highlight the importance of adjuvant treatment, with the purpose of reducing the risk of recurrence and increasing the likelihood of healing after surgical resection.

In recent years, a series of trials have evaluated the adjuvant role of interferon (IFN) as adjuvant therapy. Several schedules were investigated (high/intermediate/low-dose IFN, pegylated-IFN, ±induction), but taken together, the results were controversial, showing a limited benefit, particularly for patients with ulcerated primaries, but without confirming superior effectiveness of one specific schedule [8,9,10]. Other therapeutic options, such as anti-angiogenic agents and vaccines, have also been studied [11].

Recent advances in the understanding of melanoma biology and the success of immunotherapy, such as anti-Cytotoxic T-Lymphocyte Antigen 4 (CLTA-4) and anti-Programmed cell death protein 1 (PD-1) antibodies, and therapies targeting serine/threonine kinase (BRAF) and mitogen-activated protein kinase 1 (MEK) have led to significant prognostic improvements in unresectable stage III and metastatic stage IV CM patients [12,13,14].

In most cases, the primary endpoint in evaluating the efficacy of adjuvant therapy is disease-free survival (DFS) or relapse-free survival (RFS), defined as the time elapsed between randomization and any recurrence (local, distant) or death for any reason. However, another relevant endpoint analyzed in clinical trials and usually selected as a secondary endpoint is the distant metastasis-free survival (DMFS), defined as the time from randomization to the development of any distant metastasis or death; this endpoint effectively represents the potential efficacy of treatment in preventing metastatic development [15].

This review focuses on the impact of adjuvant treatments that are either approved and reported in international guidelines or studied in protocols and clinical trials in order to investigate whether DMFS evaluation could add additional relevance to the known endpoints for patient evaluation, thus integrating the current European Society for Medical Oncology (ESMO) guidelines on melanoma management. The main studies on adjuvant therapy for resected stage III/IV CM, including those related to positive SLN management, were analyzed with a specific focus on DMFS in either the treatment or observational/placebo arms. The aims are: to evaluate the trends of DMFS in these studies and determine whether or not they are associated with RFS; to identify which treatments are potentially associated with a better outcome in terms of DMFS and which predictive factors are related to a different outcome; and to discuss the significance of DMFS trends in terms of the clinical management of patients in daily practice.

## 2. Results of Principal Adjuvant Trials in CM

### 2.1. Interferon

The first approved adjuvant treatment for melanoma in Europe was IFN-α, but it did not entirely fulfill expectations. It was based on the hypothesis that micro-metastatic disease may induce tumor tolerance in the host and that IFN, acting on the immune system, could therefore improve the course of the disease [16].

The EORTC 18,952 trial investigated the role of intermediate-dose IFN a-2b vs. observation in 1388 stage IIb-III melanoma patients. No advantage of IFN over observation was demonstrated in patients without ulceration or in patients with stage III-N2 and ulcerated melanoma. On the contrary, a treatment effect was observed on RFS and DMFS in stage IIb/III-N1 [17]. At a median follow-up of 10 years, the impact on RFS and DMFS in non-ulcerated primaries was null, with hazard ratios (HRs) of IFN at 13 months and IFN at 25 months vs. observation of 0.94 vs. 0.84 (*p* = 0.06) for RFS and 0.95 vs. 0.84 (*p* = 0.07) for DMFS. On the other hand, in patients with ulcerated melanoma stage IIB/III-N1 (i.e., microscopic nodal involvement), the HR estimates were 0.85 vs. 0.62 for RFS and 0.80 vs. 0.56 for DMFS [9,18,19,20].

In the EORTC 18,991 trial, PEG-IFN-α-2b vs. observation in 1256 stage III melanoma patients was evaluated. Treated patients showed a significantly improved RFS compared to observation (39.1% vs. 34.6%) at a median follow-up of 7.6 years, yet no difference was found in DMFS [9]. The former group showed a median RFS of 3.0 years vs. 2.2 years in the observation arm, with a 13% reduction in the risk of recurrence or death in the treatment arm (HR, 0.87; 95% CI, 0.76 to 1.00) compared with observation (*p* = 0.055) and a 4.5% (95% CI, 0.6% to 12.8%) absolute difference in the estimated 7-year rate of RFS (39.1% in the treatment arm vs. 34.6% for observation). The RFS benefit, with an HR of 0.82, decreased to an HR of 0.87 at 7 years.

The 7-year DMFS rates were 41.7% in the PEG-IFN-α-2b arm and 40.0% in the observation arm, and this did not reach statistical significance. After subdividing patients into microscopic nodal disease (N1) and palpable nodal disease (N2), the risk of distant metastases was reduced by 14% when comparing N1 treated and untreated groups (DMFS HR 0.86; 99% CI 0.63 to 1.17; *p* = 0.22), with an improvement in the 7-year DMFS rate of 5.3%. No evidence of a benefit was observed in N2 patients. Moreover, patients with one lymph node involved showed a greater reduction in the risk of distant metastases (HR, 0.85; 99% CI, 0.63 to 1.15) compared with patients with multiple lymph nodes involved.

Furthermore, in patients with microscopic nodal involvement and tumor ulceration, the impact of PEG-IFN on DMFS was significant and sustained over time (median 4.0 vs. 2.3 years; HR, 0.65; 99% CI, 0.41 to 1.04; *p* = 0.02).

These results suggest that the impact of IFN therapy on DMFS was significant in patients with SN+ and ulcerated primary tumors. However, no benefit in DMFS or OS was observed with IFN treatment in N2 patients with bulky nodal disease (comparable to AJCC7 stage IIIB).

Similar results were obtained in the ECOG 1684 trial, where IFN alpha-2b-treated patients presented median DFS survival (from 1 to 1.7 years) and OS (from 2.8 to 3.8 years) compared to observation, and the benefit of therapy was greatest among node-positive patients [21]. Unfortunately, in this study, no data about DMFS were reported.

### 2.2. Adjuvant Trials before New Drugs

#### 2.2.1. Bevacizumab

The key driver of angiogenesis, vascular endothelial growth factor (VEGF), is over-expressed in CM, and higher levels are associated with poorer outcomes. The AVAST-M trial assessed the role of adjuvant bevacizumab (recombinant humanized monoclonal antibody directed against VEGF) in melanoma patients with resected stage IIB-C and IIIA, B and C, who received either adjuvant bevacizumab or standard follow-up [11].

Patients in the treatment arm had a higher 5-year disease-free interval (DFI) rate (51%; CI 47−55%) compared to patients in the observation arm (45%; CI 42−49%), with a median DFI for patients in the former arm of 63 months (CI, 44 months to limit not reached) vs. 37 months (CI 30–50 months) in the latter.

No significant statistical differences were observed in the 5-year DMFS, with percentages of 58% (CI 54–62%) in the treatment arm and 54% (CI 50−58%) in the observation group (HR, 0.91; CI 0.78–1.07; *p* = 0.25). In the bevacizumab arm, the median DMFS was not reached, vs. 9.6 years in the observation arm. Moreover, no significant differences in OS between treated patients and observations were observed, only a trend in BRAF+ patients (HR = 0.80; CI 0.57–1.13; *p* = 0.21) that was not seen in BRAF wild-type patients (HR = 1.17; CI 0.82–1.61; *p* = 0.34).

In this study, the predictive value of lactate dehydrogenase (LDH) levels in plasma was also investigated, and the results were not significant for either DFS, DMFS or overall survival (OS).

According to these results, bevacizumab should not be recommended as a standard adjuvant therapeutic option [11].

#### 2.2.2. MAGE-A3 (Melanoma-Associated Antigen 3)

The DERMA study evaluated the efficacy of the tumor-specific antigen MAGE-A3, which is expressed by various tumors, including melanoma, combined with an immunostimulant in order to enhance the immune response against the tumor [22].

Stage IIIB-C patients with CM and macroscopic lymph node involvement received 13 intramuscular injections of recombinant MAGE-A3 with the AS15 immunostimulant or placebo for 27 months.

The study was stopped because no treatment effect was observed, as no improvement in DFS, OS or any other clinical outcome was detected [22,23]. At a median follow-up of 28.0 months (Interquartile Range IQR 23.3−35.5) for the treatment group and 28.1 months (IQR 23.7−36.9) for the placebo group, a 64% rate of recurrence or death was found in the former group vs. 63% in the latter. At a median follow-up of 54.3 months (IQR 47.8−58.6) in the MAGE-A3 group and 54.3 months (47.0−58.1) in the placebo group, the median DFS was 11.0 months (95% CI 10.0−11.9) and 11.2 months (8.6−13.3) (HR 1.02, 95% CI 0.89−1.18, *p* = 0.75), respectively [23].

For DMFS, 743 events (502 of 893, 56%, in MAGE-A3 and 241 of 452, 53%, in placebo) were observed; the median DMFS was 18.7 months (16.3–22.1) and 23.9 months (18.9−30.7) (HR 1.09, 0.94–1.27; *p* = 0.27), respectively.

#### 2.2.3. Canvaxin^TM^ Plus Bacillus Calmette Guerin

The Malignant Melanoma Active Immunotherapy Trial in Stage IV disease (MMAIT-IV) evaluated the efficacy of an allogeneic whole-cell vaccine (Canvaxin^TM^, Cancervax, Carlsbad, CA, USA) plus bacillus Calmette–Guerin (BCG, Organon Teknika Corporation, Oklahoma City, OK, USA) after complete resection of stage IV melanoma. This approach was based on the rationale that the combination of antigens and immune adjuvants (in this case, BCG) can play an important role in enhancing the immune response against the tumor through the recruitment of macrophages, antigen-presenting cells and lymphocytes to the site of vaccine administration [24].

A total of 496 completely resected stage IV patients were included in this study and randomized to receive either BCG + Canvaxin or BCG + placebo. No more than five metastases in no more than two organs at the time of surgery were required before starting the study drug (14–90 days after surgery). The median duration of study drug administration was 8.1 months. Vaccination was stopped at recurrence but possibly restarted after completion of treatment for recurrence (except in the case of systemic chemotherapy). No statistically significant difference was found in terms of OS (median OS 38.6 in the placebo group vs. 34.9 months in the BCG + Canvaxin group). The placebo arm showed a median DFS of 7.6 months, while the BCG + Canvaxin group showed a value of 8.5 months (HR 0.88; 95% CI 0.71–1.10; *p* = 0.260). DFS was 39.9% and 43.6% at 1 year and 23.8% and 30.0% at 5 years for placebo and BCG + Canvaxin arms, respectively.

In conclusion, these results showed no benefit from adjuvant BCG + Canvaxin treatment, yet it is important to highlight that more than 40% of patients were still alive after 5 years, while in previous studies, 5-year survival following surgery alone displayed rates of around 20% [25]. This study emphasized that long-term survival can be achieved through surgical resection of metastases, as well as the potential favorable role of BCG. This is an important result, especially given the cost difference between approved drug therapies and surgical resection, which is much more cost-effective [26].

### 2.3. Immune Checkpoint Inhibitors (ICIs)

Melanoma tumor growth and progression are associated with immune suppression thanks to the ability of tumor cells to express ligands of immune checkpoint pathways. Immune checkpoint inhibitors (ICIs) are monoclonal antibodies that mainly target two pathways, CTLA-4 and PD-1.

#### 2.3.1. Ipilimumab

Ipilimumab is a human IgG1 monoclonal antibody that exerts its function by blocking CTLA-4, inducing an enhanced anti-tumoral response [27,28].

In the EORTC 18,071 trial, stage III melanoma patients were randomly assigned to receive either 10 mg/kg ipilimumab or placebo every 3 weeks for four doses and then every 3 months for up to 3 years. Patients with lymph node metastases ≤ 1 mm or in-transit metastasis were not included in the trial.

At a median follow-up of 2.74 years, RFS was significantly longer in the treatment group (HR 0.75, 95% CI 0.64–0.90; *p* = 0.0013); moreover, median RFS was 26.1 months (95% CI 19.3–39.3) vs. 17.1 months (13.4–21.6) in the ipilimumab and placebo arms, respectively. The study suggested that patients with microscopic lymph node involvement or ulcerated CM seemed to benefit from ipilimumab more than patients with palpable lymph nodes or non-ulcerated melanoma, respectively [29].

#### 2.3.2. Nivolumab

In Europe, nivolumab was approved as adjuvant melanoma therapy in 2017 [30].

The CheckMate 238 trial analyzed the efficacy of nivolumab in stage IIIB-C and IV disease-free melanoma patients. For one year, the patients received an intravenous infusion of either nivolumab or ipilimumab with a corresponding matching placebo. Collectively, these results demonstrated that nivolumab treatment improves RFS in stage IIIB-C or IV disease-free melanoma patients.

The study evidenced a significant reduction in RFS (HR 0.65; CI 97,56% 0.51–0.83). At 12, 18, 24, 36 and 48 months in the nivolumab group, values of 70%, 66%, 62%, 58% and 52% were calculated, respectively, while for the ipilimumab group, they were 61%, 53%, 51%, 45% and 41%, respectively.

At 12 months, DMFS was 80% in the nivolumab arm and 73% in the ipilimumab arm, with an HR of 0.76 (CI 95% 0.59–0.96).

Higher DMFS was observed in the nivolumab group at a follow-up of 24, 36 and 48 months [31], while the median DMFS was not reached in either treatment group.

At 36 months, DMFS was 66% in the nivolumab group and 58% in the ipilimumab group.

At 48 months of follow-up, DMFS was still more favorable in the former arm (59% vs. 53%) (HR 0.79; 95% CI 0.63–0.99) [28].

Similar results were observed for RFS in the subgroup of BRAF-mutated and wild-type patients.

At the 2021 American Society of Clinical Oncology (ASCO) congress, James Larkin et al. presented an analysis of patients with in-transit metastases (ITM) treated with either nivolumab or ipilimumab in CheckMate 238. This study included patients with ITM with and without nodal involvement, unlike adjuvant studies with ipilimumab and pembrolizumab that excluded these patients and with dabrafenib plus trametinib that included very few [32,33].

Nivolumab demonstrated an RFS benefit vs. ipilimumab in patients with and without ITM. In particular, the benefit was more prominent in patients with ITM than in those without ITM (HR, 0.63 vs. 0.77), especially in patients with nodal involvement (HR, 0.57; 95% CI, 0.37–0.87) compared to those without nodal involvement (HR, 0.72; 95% CI, 0.47–1.12). Similar to RFS, nivolumab demonstrated improved DMFS vs. ipilimumab in the ITM subgroups.

Similar metastasis patterns were observed in patients with ITM, regardless of nodal involvement, and in patients without ITM [34].

#### 2.3.3. Nivolumab Plus Ipilimumab

The CheckMate 915 trial compared the clinical activity of combined ipilimumab and nivolumab as adjuvant treatment vs. nivolumab alone in stage IIIB-D/IV patients.

This study was the first that did not require patients to undergo CLND and in which the stage was assigned according to the 8th AJCC classification. Patients were randomized to receive an infusion of nivolumab (dose 240 mg every 2 weeks) + ipilimumab (dose 1 mg/kg every 6 weeks) or nivolumab alone (dose 480 mg every 4 weeks).

At 24 months, RFS rates were 64.6% in the combination treatment group vs. 63.2% in the nivolumab group (HR 0.92; CI 97.295, 0.77–1.09), with no relevant difference between stage III and IV subgroups (64.7% and 63.6% vs. 63.6% and 61.1%, respectively, for nivolumab + ipilimumab and nivolumab in stages III and IV).

At 24 months, stage III patients showed a DMFS of 75.4% in the combination treatment group and 77.4% in the nivolumab group (HR 1.01, CI 95% 0.83–1.23), and in the subset of stage III PD-L1 < 1%, it was 68.4% and 67.9% in the combination and nivolumab groups, respectively (HR 0.94; CI 0.70–1.25).

Therefore, this study did not confirm the superiority of the combined treatment compared to nivolumab alone. However, it should be emphasized that the schedule of the combination was different from the classic one used in the CheckMate 067 trial. In this trial, patients received nivolumab 1 mg/kg every 3 weeks + ipilimumab 3 mg/kg every 3 weeks (for four doses), followed by nivolumab (3 mg/kg every 2 weeks), or nivolumab 3 mg/kg every 2 weeks or ipilimumab 3 mg/kg every 3 weeks (for four doses) [33,35].

#### 2.3.4. IMMUNED

This study aimed to evaluate the efficacy and safety of adjuvant combination immunotherapy with ipilimumab + nivolumab (combination) vs. nivolumab alone vs. placebo in stage IV with no evidence of disease (NED) patients. In total, 167 patients were included, with a 1:1:1 randomization in the three different arms.

In the combination arm, RFS was 75% (95% CI 61.0–84.9) at 1 year and 70% at 2 years; in the monotherapy arm, RFS was 52% (38.1–63.9) at 1 year and 42% at 2 years; in the placebo arm, RFS values were 32% (19.8–45.3) and 14% at 1 and 2 years, respectively.

The hazard ratio for recurrence for combination vs. placebo was 0.23 (97.5% CI 0.12–0.45; *p* < 0.0001), and for monotherapy vs. placebo, it was 0.56 (0.33–0.94; *p* = 0.011).

Overall, a remarkable prolongation of RFS was observed for adjuvant use of ipilimumab + nivolumab compared with nivolumab alone or placebo.

For nivolumab, the observed RFS rates (52% at 1 year, 42% at 2 years) were lower than those of stage IV-NED melanoma (63% at 1 year, 58% at 2 years) in CheckMate 238. However, as reported by the authors, fewer stage M1a patients (39% vs. 61%) and more stage M1c patients (31% vs. 34%) were enrolled in this trial.

#### 2.3.5. Pembrolizumab

The European Organization for Research and Treatment of Cancer (EORTC) 1325/KEYNOTE-054 trial evaluated treatment with pembrolizumab compared to placebo in stage IIIA (with at least one micro-metastasis >1 mm), B and C melanoma patients with resected high-risk melanoma. In the case of recurrence (except encephalic metastases), patients who had received the placebo were eligible for crossover. Rechallenge with pembrolizumab for up to 2 years was allowed in patients previously treated in the pembrolizumab arm (with disease recurrence at least six months after completing one year of therapy).

At both 15 months (HR 0.57; CI 98.4%, 0.43–0.74; *p* < 0.0001) and 3 years (63.7% vs. 44.1%; HR, 0.56; 95% CI, 0.47–0.68) of follow-up, adjuvant treatment with pembrolizumab improved RFS vs. placebo [36,37].

These results confirmed that pembrolizumab provided a remarkable improvement in RFS [38].

In the intention to treat (ITT) population, at 1, 2, 3 and 4 years, the DMFS rate was 82.8%, 73.5%, 68.2% and 65.3% in the pembrolizumab group and 69.8%, 56.0%, 51.5% and 49.4% in the placebo group, respectively.

The DMFS was significantly longer in the pembrolizumab group than in the placebo group (HR 0.60; 95% CI 0.49–0.73, *p* < 0.0001). This was also observed in patients with PD-L1-negative and -positive melanomas and across subgroups according to baseline characteristics (sex, age, ulceration, BRAF mutation status, lymph node involvement), similar to that in the ITT population (also between patients with both AJCC-7 and AJCC-8 staging). With regard to the comparison between the various subgroups in this study, a more specific statistical test, an iterative forest plot, was used to ensure that the results were indeed adequate [37].

DMFS values were significantly lower in the PD-L1-negative subset in both pembrolizumab and placebo groups (58.0% and 40.2%, respectively), compared to the same values in the PD-L1-positive subgroup (66.7% and 51.6%, respectively). These results show that PD-L1 expression represents a prognostic rather than a predictive biological marker.

In addition, the study also identified a different pattern of disease relapse between pembrolizumab-treated patients and the placebo group. Indeed, treatment with pembrolizumab was found to be more effective in reducing the onset of distant metastasis as the first event of relapse and in reducing the percentage of loco-regional recurrence alone (39.5% and 24.9% distant metastasis as the first event in placebo and pembrolizumab groups, respectively: 18% and 14%, loco-regional recurrence alone in placebo and pembrolizumab groups, respectively) [37].

As reported at ASCO 2021, both crossover and rechallenge in patients with recurrence show interesting results. In particular, pembrolizumab treatment after crossover shows a median PFS of 8.5 months and a 3-year PFS of 32%, while rechallenged patients show shorter median PFS (4 months) and 1-year PFS of ~40%.

The safety profile was favorable and comparable to that observed in the adjuvant trials.

In the Southwest Oncology Group (SWOG) S1404 trial, 1303 patients with high-risk resected melanoma (AJCC 7 Stage IIIA[N2a] to stage IV or relapsed stage III disease) were randomized to receive pembrolizumab or “standard of care” (high-dose interferon or ipilimumab).

For RFS, pembrolizumab-treated patients showed better results at 3.5 years of follow-up, with an HR (99.62% CI) of 0.74 (0.57, 0.96) *p*-value < 0.001, but the same cannot be said for OS (HR 0.84, [0.62–1.13]). The safety profiles of pembrolizumab, ipilimumab and high dose interferon (HDI) are similar to those in previous adjuvant studies.

This trial evidenced that single-agent anti-PD1 antibody treatment should be a standard of care option in the adjuvant setting [39].

### 2.4. Targeted Therapy

Cell survival and proliferation signals from the membrane to the nucleus require certain proteins for their correct transduction, one of which is encoded by the BRAF gene. Its mutation results in the presence of an altered protein, found in approximately 50−60% of melanoma patients, which provides the tumor with an externally independent proliferative stimulus, making it more aggressive and more proliferative.

These findings have led to the development of specific inhibitors [40].

#### 2.4.1. Dabrafenib + Trametinib

Dabrafenib (BRAF inhibitor) and trametinib (MEK inhibitor) target the mitogen-activated protein kinase pathway and have been used in combination for metastatic melanoma. Their use was also approved for the adjuvant setting in 2018 [41].

In the phase III COMBI-AD trial, 870 patients with stage IIIA (lymph-node metastasis > 1 mm), B and C (AJCC 7th edition [42]) with proven BRAF mutation (either V600E or V600K) were randomized to receive either treatment with the two oral drugs or placebo. They were stratified according to their stage and treated for 12 months [32].

Patients were evaluated at a median follow-up of 2.8 years. A 53% statistically significant lower risk of relapse (HR 0.47; 95% CI, 0.39 to 0.58; *p* < 0.001) was found in the treatment group. RFS rates were 88%, 67%, 58%, 55% and 52% at 1, 2, 3, 4 and 5 years in the targeted therapy arm vs. 56%, 44%, 39%, 38% and 36% at 1, 2, 3, 4 and 5 years in the placebo group. Higher rates of DMFS compared to placebo, with a clinically meaningful lower risk of 49%, and a consistent benefit of combination therapy across all subgroups of patients in the analysis were described.

The benefit of the combination treatment in DMFS was observed in every year of follow up: 91% vs. 70% after one year, 77% vs. 60% after two years, 71% vs. 57% after three years, 67% vs. 56% after four years and 65% vs. 54% after five years (HR 0.55; 95% CI, 0.44 to 0.70), with durable DMFS improvement in the combination therapy group with extended follow-up [43]. This benefit was observed across stages (stages IIIA, IIIB and IIIC patients), and in all of these subgroups, the median value was reached in the combination therapy arm.

An important consideration to be evaluated in this trial is that, according to protocol procedures, patients whose first relapse was loco-regional were not required to continue follow-up for distant metastases, and their data were censored for the DMFS analysis. On the other hand, in the pembrolizumab study, distant metastases after loco-regional recurrence were also counted as events [37]. As pointed out by Eggermont et al., such information censoring could lead to an incorrect estimation of the percentages of patients with distant metastases and might bias the comparison. As a reply reported in a commentary by Georgina V. Long et al., this study modality would most likely have favored the placebo group, since there were more patients who first presented with local or regional recurrence in the placebo group, and this would mean that the benefit regarding DMFS presented in the article might actually be underestimated [44].

#### 2.4.2. Vemurafenib

In the BRIM8 study, the adjuvant vemurafenib was evaluated in resected stage IIC-IIIA, IIIB and IIIC (BRAF V600+) melanoma patients. For 52 weeks, patients received either vemurafenib (twice a day) or placebo. Patients were subdivided into two cohorts (cohort 1 included stage IIC, IIIA and IIIB patients and cohort 2 included stage IIIC patients). The median follow-up was 33.5 months in cohort 2 and 30.8 in cohort 1.

In cohort 2, median DFS was 23.1 months (95% CI 18.6–26.5) in the treatment arm vs. 15.4 months (11.1–35.9) in the placebo arm (HR 0.80, 95% CI 0.54–1.18; *p* = 0.26), with a 1-year DFS of 78.9% (95% CI 70.5–87.3) in the former arm vs. 58.0% (95% CI 47.8–68.1) in the latter. The 2-year DFS was 46.3% (95% CI 35.4–57.1) vs. 47.5% (95% CI 37.1–57.9), respectively.

In cohort 1, median DFS was not reached (95% CI not estimable) in the treatment arm vs. 36.9 months (21.4–not estimable) in the placebo arm (HR 0.54; 95% CI 0.37–0.78; *p* = 0.0010). However, because of the hierarchical prerequisite for the primary DFS analysis of cohort 2 to show a significant DFS benefit, this result should be considered exploratory only.

In cohort 2, the median DMFS was 37.2 months (95% CI, 22.1-NE) in the vemurafenib group vs. 30.7 months (24.5-NE) in the placebo group (HR 0.91, 95% CI 0.57–1.44, *p* = 0.68); at 1 year and 2 years, the percentage of patients alive without distant metastasis was 83.2% vs. 77.0% and 57.5% vs. 62.4%, respectively (HR = 0.91; 95% CI 0.57–1.44; *p* = 0.68).

In cohort 1, the median DMFS was not reached in either treatment group (HR for vemurafenib vs. placebo 0.58, 95% CI 0.37–0.90, *p* = 0.013); at 1 year, the percentage of surviving patients without distant metastasis was 88.9% and 79.5% in the vemurafenib and placebo groups, respectively, and at 2 years, the corresponding values were 81.0% and 66.9%, respectively (HR = 0.58; 95% CI 0.37–0.90; *p* = 0.013).

Combining cohorts 1 and 2, the percentage at 1 year was 86.7% and 78.5%, respectively, in the vemurafenib and placebo groups, and at 2 years, it was 72.0% and 65.3%, respectively (HR = 0.70; 95% CI 0.52–0.96; *p* = 0.027), but these data should be considered exploratory only [45].

## 3. CLND or Nodal Observation in SLN-Positive Patients: Does It Have an Impact on DMFS?

### 3.1. DECOG-SLT

In this trial, DMFS was the primary endpoint, with the aim of evaluating whether CLND improved survival in patients with sentinel lymph node (SLN) metastases. A total of 5547 patients with a diagnosis of CM of at least 1 mm in thickness and micro-metastasis in the SLN (including single cells) were screened. Patients with melanoma of the head and neck region or with satellite, in-transit, distant metastasis or regional macro-metastasis were excluded. Of the enrolled patients, 1269 were SLN positive, 473 of which were randomized to CLND (242) or nodal observation with nodal basin ultrasound every 3 months.

The median patient age was 54 years, and more than half of the patients (60%) had a tumor thickness greater than 2 mm (2.4 mm in both groups). In 435 out of the 473 patients included in the study, only one SLN was positive. The median follow-up time was 35 months.

Distant metastases were recorded in 85 out of 473 patients (18%), with similar percentages in both groups (43 in the observation arm vs. 42 in the CLND arm) and in 31 out of 54 patients (57%) with regional lymph node metastases (19 in the former group vs. 12 in the latter). Moreover, similar DFMS rates were found at 3 years: 77% (90% CI 71.9–82.1; 55 events) vs. 74.9% (69.5–80.3; 54 events), with a hazard ratio of 1.03 (90% CI 0.71–1.50, *p* = 0.87). In the article, the authors reported that tumor load in the SLNB and tumor thickness were significant predictors of RFS, OS and DMFS, whereas CLND vs. observation had no significant effect on survival.

Interestingly, after surgical intervention, 24% of patients in the CLND group developed adverse events. Grade 3–4 events occurred in 6% and 8%, respectively.

Although the study was closed early, it highlighted that there was no significant difference in 3-year DMFS, RFS or OS between the CLND arm and the observation arm [46]. It is important to note that most patients (311; 66%) had a low tumor burden in SLN, not exceeding a diameter of 1 mm. Based on these findings, CLND should not be recommended in patients with micro-metastases, at least in those with single cells or micro-metastases 1 mm or smaller. Overall, as there are no differences in adjuvant systemic therapy based on the number of positive lymph nodes, CLND appears to be dispensable in these patients [47].

### 3.2. MSLT II

In this trial, patients with SLN metastases were randomized to undergo immediate CLND or observation with ultrasonography.

At 3 years, no significant difference in melanoma-specific survival (86 ± 1.3% and 86 ± 1.2%, respectively; *p* = 0.42) was found between the two arms. DFS was slightly higher in the CLND group (68 ± 1.7% vs. 63 ± 1.7%; *p* = 0.05), probably due to a reduced rate of nodal recurrence following CLND, with increased regional disease control in the CLND group (92 ± 1.0%) compared to observation (77 ± 1.5%, *p* < 0.001). However, no significant benefit in melanoma-specific survival was found in any groups in a subgroup analysis. Moreover, almost one out of four patients experienced lymphedemas after CLND vs. only 6.3% in the observation group. For DMFS, no statistically significant difference was found between the two groups (HR, 1.10; 95% CI, 0.92 to 1.31; *p* = 0.31).

Melanoma thickness (Breslow) and the number of positive SLNs on pathological assessment were found to be significant prognostic factors in both groups, whereas the male sex was significant only in the observation group.

An analysis performed only on patients with positive SLNs on pathological assessment (i.e., excluding RT-PCR-positive lymph nodes) showed that the number of involved SLNs and male sex were no longer significant prognostic factors in either group. On the contrary, melanoma thickness remained a significant prognostic factor in both groups, while the pathological status of non-SLN was significant in the CLND group.

In the observation group, the detection rate of non-SLN involvement exceeded the percentage in the CLND group both at 3 years and at 5 years (*p* = 0.02 and *p* = 0.005, respectively).

In this study, the pathological status of non-sentinel nodes was proved to be a valid independent prognostic factor; on the contrary, the overall number of involved sentinel nodes was not found to be significantly related to melanoma-specific survival. Moreover, while CLND increased regional disease control, providing prognostic information, it did not increase melanoma-specific survival among patients with SLN metastases [48].

In MSLT-I, patients with nodal disease and intermediate-thickness melanomas had better outcomes with immediate compared to delayed CLND; in MSLT-II, the lack of survival benefit with CLND suggests that any increase in OS with immediate CLND occurred among patients with disease limited to the SLN, while patients with non-SLN metastases may still benefit from salvage CLND, but the timing of that intervention does not seem to be critical [49,50].

## 4. Safety Profile

Although these treatments are effective, they carry a demonstrated risk of adverse effects (like any drug), both for targeted therapy and immunotherapy. Although, in most cases, these side effects are mild or moderate, their impact on a patient’s life can be significant and should not be underestimated, especially as the new IIIa stage patients present a 3yOs of 83.1% [51].

### 4.1. Interferon

In the EORTC 18,991 trial, according to NCI-CTC classification, a grade 3 event was seen in 57% of patients treated with PEG-IFN (14% in the observation arm), and 9% of the treated arm evidenced a grade 4 event (4% in observation arm) [9].

### 4.2. Immunotherapy

Adverse events of any cause were recorded in 96.9% in the nivolumab group and 98.5% in the ipilimumab group, while serious adverse events were 17.5% vs. 40.4%, and drug discontinuation was reported in 9.7% vs. 42.6%, respectively.

Ipilimumab has a worse tolerance compared with anti-PD-1 and targeted therapies [52], with percentages of grade 3–4 immune-related adverse events of 41.6% vs. 2.7% in the placebo arm [5,23,53]. Nivolumab has shown less severe toxicity compared with ipilimumab [28].

In the IMMUNED trial, grade 3–4 adverse events were seen in 71% (95% CI 57–82) of patients in the combination group and in 27% (16–40) of patients treated with single-agent nivolumab.

In the pembrolizumab group, 77.8% of patients showed adverse events of any grade, whilst 66.1% was observed in the placebo group. In a study on pembrolizumab, it was shown that the onset of immune toxicity was related to a higher survival rate, which would lead to the assumption that treating a subject with a lower risk of toxicity would involve the risk of less effective treatment [54,55].

For anti-PD-1, pembrolizumab and nivolumab show similar adverse events in both the adjuvant and metastatic settings. Immune-related adverse events were seen in 37% of patients (grades III-IV in roughly 15% of patients). Endocrine toxicities are quite common in both treatments and often irreversible (thyroid toxicity 15−20%; hypophysitis 2.2%; diabetes mellitus 1%).

### 4.3. Targeted Therapy

The most common adverse events were pyrexia (5% grades 3–4), fatigue (4% grades 3–4) and nausea (<1% grades 3–4). Serious AEs were seen in 36% in the combination therapy group and 10% in the placebo group; adverse events led to discontinuation of a trial drug in 26% of patients and to dose reduction in 38%.

For vemurafenib, 20% of patients discontinued treatment due to adverse events, while most reported adverse events were easily manageable (grades 1–2).

The safety profile of dabrafenib plus trametinib was similar in both metastatic and adjuvant settings [23]. In the COMBI-AD trial, 26% of patients discontinued treatment due to adverse events (this figure, higher than those reported in studies on metastatic melanoma, could be due to patient- or physician-related reasons or to different schedules of pyrexia management) [32].

## 5. DMFS According to BRAF Mutation in Adjuvant Trials

The prognostic relevance of BRAF mutation is still a matter of controversy, and based on present data, no definite conclusion can be drawn. However, some data from adjuvant trials can be considered to gain more insights into this debated topic. Before the new-therapy era, the AVAST-M study, which did not meet its principal endpoint, as it did not demonstrate any advantage in the drug arm with bevacizumab, showed a significantly lower OS of BRAF+ patients compared to BRAF WT patients [11].

In the CheckMate 238 trial, RFS in BRAF-mutated patients at 36 and 48 months was 56% and 52% in the nivolumab arm and 47% and 44% in the ipilimumab arm vs. 58% in nivolumab-treated and 42% in ipilimumab-treated groups at 36 months (50% vs. 39% at 48 months) in wild-type patients [22].

In the IMMUNED trial, patients with positive BRAF mutation seemed to particularly benefit from combined immunotherapy (ipilimumab + nivolumab), whereas no difference according to the BRAF mutation pattern was found in the other two treatment arms, but the reason for this is not fully understood, and conflicting data exist [56].

The analysis of patients included in trials with targeted or immunotherapy is weighted by several biases related to both the different patient inclusion criteria and the number of BRAF patients enrolled. With these limits, some interesting considerations can be drawn, as summarized in Table 1.

In KEYNOTE-054, when stratifying enrolled patients according to BRAF status, treatment with pembrolizumab led to comparable results (RFS of 62% and 61.8% at 36 months in mutated and wild-type, respectively), whereas the two placebo groups showed different trends (37.1% and 46.5% at 36 months in mutated and wild-type, respectively) with a significantly worse prognosis for BRAF-mutated patients.

DMFS analysis showed similar results. At 42 months, BRAF-mutated and wild-type patients treated with pembrolizumab showed a DMFS of 63.7% and 62.1%, respectively; on the other hand, in placebo-treated patients, the DMFS was 43.4% in the BRAF mutant group and 51.4% in the wild-type group. Therefore, considering BRAF mutant and wild-type subgroups, we can observe that, in the KEYNOTE-054 study, placebo-treated patients carrying BRAF mutations showed worse DMFS and RFS compared to wild-type patients.

However, a similar trend was not confirmed in the COMBI-AD trial, which enrolled only BRAF-mutated patients and therefore analyzed a significantly higher number of BRAF-mutated patients (870 vs. 440). In the COMBI-AD trial, the 4-year DMFS in the treated arm was 67% (thus similar to that of the KEYNOTE-054 study), whilst the values in the placebo group were higher than those in the KEYNOTE-054 study (56%), therefore not confirming a worse disease outcome.

Moreover, considering the CheckMate 238 study, no significant differences according to a BRAF mutation pattern could be demonstrated between nivolumab and ipilimumab, with even slightly higher values for mutants compared to wild-type for either RFS or DMFS.

In conclusion, the data produced by the clinical trials do not support the role of BRAF mutation as predictive of a different outcome. Future data are needed to obtain more evidence on this controversial topic. However, as far as is conceivable based on available data, it is presumable that the differences reported in some studies (i.e., KEYNOTE-054) could be related to a different distribution of clinical-pathologic features.

## 6. Discussion

Table 2 summarizes the results in terms of DMFS at different follow-up time points and according to the HRs based on the reported data in the analyzed adjuvant trials.

Although performing a direct comparison between these studies would not be methodologically accurate due to differences in the time of study development, trial design, patient accrual, stage system and patients’ characteristics, some interesting points are noteworthy.

As a general consideration, the trends of DMFS closely follow those of RFS, thus meaning that this endpoint could be considered in addition to RFS as a primary endpoint for adjuvant trials; moreover, as it captures the burden of patients who develop distant metastases and not a loco-regional relapse, this could provide a more adequate measure of patients whose relapse is associated with a worse prognosis and who need further systemic treatment. Lower HRs for DMFS were reported in the COMBI-AD (0.51, 95% CI 0.4–0.65) and KEYNOTE-054 (0.53; 95% CI 0.37–0.76) trials, which were characterized by the same inclusion criteria for patients (IIIA more than 1 mm diameter sentinel node biopsy deposit, IIIB and IIIC). The finding that targeted therapy and immunotherapy achieved the same HR reduction testify to the evidence of substantially similar disease activity of these two regimens at the follow-up times analyzed so far. When considering the trends in DMFS over time, however, it appears that the values of the COMBI-AD trial at 1 year are slightly higher than those of pembrolizumab, supporting the early activity of the targeted therapies, whereas over time, this difference does not appear to be confirmed, and the values are similar (at 4 years, values were 67.0% in COMBI-AD and 65.3% in KEYNOTE-054). CheckMate 238 obtained lower HR (0.73; 95% CI 0.55–0.95); however, it was developed with two treatment arms (nivolumab and ipilimumab) without an observation arm as in the previous study. A recent paper analyzed the potential values of HR in CheckMate 238, considering whether this study would have been conducted with a comparator observation: it showed that nivolumab would have a significant RFS benefit over observation, with an HR value of 0.54 (95% CI 0.41–0.69), similar to those of COMBI-AD and KEYNOTE-054 [57].

Looking at the comparison between “old” treatments (IFN 13 or 25 months/PEG-IFN/bevacizumab/MAGE-3A) and “new” treatments (immuno/targeted therapy), despite the differences in patient characteristics, HR is very much in favor of the latter; in particular, all trials involving old drugs show a value close to 1 or include the value 1 in the CI.

For ipilimumab, the first immunological drug tested in an adjuvant setting, the HR value showed an improvement over the previous treatments (0.76; 95% CI 0.64–0.92), but the best results were obtained with nivolumab, pembrolizumab and the combination targeted therapy. The small amount of data on DMFS, especially for IFN and ipilimumab, makes it difficult to compare results accurately. However, some observations can be made. With regard to the first 3 years of follow-up, the only data we can observe are those from the most recent studies, which show important results for the combination targeted therapy and pembrolizumab compared with placebo (91% and 82.8% vs. 70% and 69.8% at 1 year). In the first 3 years of follow-up, the benefits of these treatments compared to placebo remain stable. From the fourth year onwards, while the difference between IFN treatment and placebo decreased (48.2% vs. 45.4% at 4 years, 41.7% vs. 40% at 7 years and 33.8% vs. 32.1% at 10 years), the advantage of the new treatments in the treated groups was maintained over time, with values of 65.3% vs. 49.4% for pembrolizumab, 48.3% vs. 38.9% for ipilimumab and 65% vs. 54% for the combination targeted therapy at 5 years. The new therapies definitely show better results; at 4 years, the percentage of DMFS in the 13-month IFN-treated group was 42.3%, while the same value in the ipilimumab-treated group was 48.3% and 65% in the combination targeted group. The targeted therapy appears to guarantee a better disease outcome in the adjuvant setting in the first year of FU (91% vs. 82.8% of pembrolizumab), and then the values of DMFS approach the percentages of pembrolizumab over time, reaching 67% vs. 65.3% at 4 years.

For the placebo or observation groups (Table 3), the values have not changed over the years, and surgery has therefore remained superimposable in terms of results. Hopefully, further studies will provide the scientific community with new results, which will help to understand the impact of changes regarding CLND. The CheckMate 915 trial, in which patients did not undergo CLND, appeared to show a similar 2-year RFS in the nivolumab group (63.2%) to that in the nivolumab group of the CheckMate 238 trial (CLND performed; 63%), but the results were not statistically significant. Furthermore, in the PD-L1 subgroup <1% in CheckMate 915, the 2-year rates were 67.9%, which is comparable to CheckMate 238.

The only placebo group that appeared to have a slightly different trend to the others was that in the COMBI-AD studies. These patients seemed to have similar values to the others in the first years (70% in the first year) and then had a better trend in the following years (57% vs. 51.5% at 3 years, 56% vs. 45.4−49.4% at 4 years and 54% vs. 40−38.9% at 5 years).

**Table 2:** RFS and DMFS of the treatment arm(s) in the principal studies of adjuvant therapies/SLNB.

Study	Treatment	Inclusion	Nopts	Results															
				RFS								DMFS							
				HR	95% CI	1 yr	2 yr	3 yr	4 yr	5 yr	10 yr	HR	95% CI	1 yr	2 yr	3 yr	4 yr	5 yr	10 yr
**EORTC 18,952** [18]	IFN 13mo vs. Obs	**IIb-III**	1388	0.94	0.75–1.17 ^#^						29.3%	0.95	0.77–1.18 ^##^					42.3% ***	33.8%
	IFN 25mo vs. Obs			0.84	0.67–1.06 ^#^						33%	0.85	0.68–1.05 ^##^					46.1% ***	38.9%
**EORTC 18,991** [20]	PEG-IFN-α-2b vs. Obs	III (>1 mm)	1258	0.84	0.73–0.98				45.6%		39.1% §	0.88	0.75–1.03				48.20%		41.7% §
**AVAST-M**	Bevacizumab vs. Obs	IIB-C-III	1320	0.85	0.74–0.99	77%				51%		0.91	0.78–1.07					58%	
**DERMA**	MAGE-3A vs. Placebo	IIIB-C	1345	1.01	0.88–1.17	47%	37%	33%	31%			1.09	0.94–1.27		44%				
**EORTC 18,071** [29]	Ipilimumab vs. Placebo	III (>1 mm)	951	0.75	0.64–0.90	63.5%		46.5%		40.8%		0.76	0.64–0.92					48.3%	
**CheckMate 238**	Nivolumab vs. Ipilimumab	IIIB-C-IV NED	906	0.66	0.53–0.81	70.5% vs. 60%	63% vs. 50%	58% vs. 45%	52% vs. 41%			0.73	0.55–0.95	80% vs. 73%	70% vs. 64%	66% vs. 58%	59% vs. 53%		
**CheckMate 915**	Nivolumab + Ipilimumab vs. Nivolumab	IIIB-D-IV NED (no CLND)	1844	0.92	0.77–1.09		64.6% vs. 63.2%					1.01	0.83–1.23		75.4% vs. 77.4%				
**KEYNOTE-054** [37]	Pembrolizumab vs. Placebo	III (>1 mm)	1019	0.57	(98.4%) 0.43–0.74	75.3%	68%	63.7%	59.8% **			0.53	0.37–0.76	82.8%	73.5%	68.20%	65.3% ** [37]		
**BRIM8**	Vemurafenib vs. Placebo	IIC-IIIA/B	314	0.54	0.37–0.78	84.3%	72%					0.58	0.37–0.90	88.9%	81%				
		IIIC	184	0.8	0.54–1.18	78.9%	46%					0.91	0.57–1.44	83.2%	57.5%				
**COMBI AD** [43]	Dabrafenib + Trametinib vs. Placebo	III (>1 mm)	870	0.49	0.40–0.59	88%	67%	58%	55%	52%		0.51	0.40–0.65	91%	77%	71%	67%	65%	
**DeCOG-SLT**	CLND vs. Obs	SLNB +	483	0.95	0.72–1.25			66.8%				1.03	0.71–1.50			74.9%			
**MSLT-II**	CLND vs. Obs	SLNB+	1939					68 ± 1.7%											

Abbreviations: RFS = relapse-free survival; DMFS = distant metastasis-free survival; HR = hazard risk; IFN = interferon; SLNB = sentinel lymph node biopsy; Obs = observation; mo = months; CLND = complete lymph node dissection; ^#^ 99% HR; ^##^ 97.5% HR; ** = 3.5 years; *** = 4.5 years; § 7-year follow-up.

**Table 3:** RFS and DMFS of the observation/placebo arm(s) in the principal studies of adjuvant therapies/SLNB.

Study	(Placebo/Observation)													
	RFS							DMFS						
	1 yr	2 yr	3 yr	4 yr	5 yr	7 yr	10 yr	1 yr	2 yr	3 yr	4 yr	5 yr	7 yr	10 yr
EORTC 18,952 [18]							27.4%					40% ***		32.1%
EORTC 18,991				38.9%		34.6% [20]					45.4%		40% [20]	
AVAST-M	70%				45%							54%		
DERMA	47%	39%	35%	33%					47%					
EORTC 18,071	53.1%		34.8%		30.3% [29]							38.9%		
KEYNOTE-054 [37]	60%	46.9%	43.5%	41.4% **				69.8%	56%	51.5%	49.4% **			
BRIM8	66.2%	56.5%						79.5%	66.9%					
	58%	47.5%						77%	62.4%					
COMBI AD	56%	44%	39%	38%	36% [43]			70%	60%	57%	56%	54%		
DeCOG-SLT			67.4%							77%				
MSLT-II			63 ± 1.7%											

Abbreviations: ** = 3.5 years; *** = 4.5 year.

## 7. Conclusions

The results of this review clearly emphasize the impressive number of adjuvant trials with new drugs or combinations that have been carried out in recent years. Taken together, the results of these trials demonstrate a significant benefit for both immunotherapy with anti-PD1 (nivolumab, pembrolizumab) and targeted therapies (dabrafenib/trametinib) in the adjuvant setting in patients with stage III and/or stage IV (limited to nivolumab) melanoma patients.

The limits of these studies compared with daily clinical practice are represented by the different stage systems used (AJCC 7 in clinical trials vs. AJCC 8 in daily practice) and by the different surgical treatments of the nodal regional basin (CLND in most trials vs. observation only in most SLNB-positive patients in daily practice). Moreover, as most of the abovementioned trials have been carried out in North America and Europe, the role of adjuvant therapies in Asians’ cutaneous melanomas (which more commonly present as ALM or nodular melanoma) may not have been thoroughly investigated, and further studies should take this topic into account [58].

A second point is represented by the lack of effective adjuvant treatments for stage II melanoma patients with negative SLNB. Apart from interferon, which was shown to have a limited impact, mostly in patients with ulcerated primaries, there are no data for either immunotherapies or targeted therapies in these patients. The results of studies that have been recently concluded or are still ongoing will be extremely useful in gaining more insights into this topic (NivoMela and CheckMate 716 are trying to evaluate the efficacy of nivolumab in stages IIB and C). At the ESMO congress in September 2021, the CheckMate 716 interim analysis on pembrolizumab in the adjuvant setting for resected stage IIB-IIC melanoma reported that the risk of disease recurrence or death decreased by 35% compared with placebo, with prolonged RFS and a favorable benefit–risk profile. These promising results may indicate that these stage II patients could also benefit from adjuvant immunotherapy, making its approval likely in the near future [59]. Another study with the targeted therapy encorafenib/binimetinib is currently ongoing [60].

Despite the difficulties in comparing the results of studies regarding anti-BRAF + anti-MEK and anti-PD-1, both demonstrated a significant increase in DMFS.

For immunotherapy, the studies evaluated in this review show better values in terms of DMFS in patients treated with pembrolizumab or nivolumab, with values showing greater efficacy at the fourth year of follow-up (65.3% vs. 59%, respectively), compared to ipilimumab or placebo.

CheckMate 915 showed no significant difference in patients treated with Nivo + Ipi compared to nivolumab only. No studies have compared nivolumab to pembrolizumab. Targeted therapy with dabrafenib + trametinib shows significant results (at 5 years of follow-up, the value is about 65%). The comparison of data achieved with immunotherapy and targeted therapy shows overall similar results in terms of DMFS: 4-year DMFS was 67% in the COMBI-AD trial and 65.3% in the KEYNOTE-054 trial, which had similar inclusion criteria; at 5 years, only COMBI-AD data are available (65% DMFS). The DMFS rates of CheckMate 238 are slightly lower (58%), but it must be underlined that this trial included patients with worse predictive factors and higher stages (IIIB, IIIC-IV NED). For all of these treatments, there are no data beyond 5 years; the only data at 7 and 10 years were reported by EORTC 18,952 and 18,991 for interferon vs. placebo, showing a non-significant difference between treatment and placebo. Therefore, it remains essential to continue the follow-up. Surrogate endpoints have generally been used in oncology to swiftly assess the efficacy of potential treatments, whereas OS analyses often require extended follow-up and can be confounded by subsequent lines of treatment [61]. In the past years, several studies have evaluated the correlation between OS and surrogate endpoints in different tumor types. For melanoma patients, PFS has been shown to be an acceptable surrogate for OS [62,63]. However, as seen in CheckMate 238 and SWOG S1404 trials, RFS and OS are not always related: in fact, while better RFS results were observed in both anti-PD1 groups, no statistically significant improvement in OS was found in either of the two trials. Therefore, it is reasonable to believe that longer follow-ups are essential to fully evaluate the efficacy results of new adjuvant treatments in terms of improved survival. As stated by Suciu et al., in fact, the evaluation of efficacy results in terms of RFS, along with the safety profile, should continue to properly appraise the value of new adjuvant melanoma treatments, while overall survival evaluation, performed later, should support the initial RFS findings [61].

Overall, the analysis of DMFS shows that this endpoint follows a similar trend to RFS, and thus, a benefit in RFS is associated with a benefit in DMFS. Moreover, comparing DMFS and RFS, it appears clear that the majority of patients relapse at visceral compared to regional sites. DMFS identifies patients who need a second line of systemic treatment (first as metastatic, second after adjuvant). The significant reduction in the percentage of patients who develop distant metastases compared to previous data, either with interferon or only observational follow-up, carries a two-fold significance. On the one hand, this determines an improvement of the disease outcome in patients treated with new drugs in the adjuvant setting and significantly reduces the number of patients who need systemic treatment for their metastatic disease. On the other hand, however, it is associated with a change in the characteristics of patients who receive systemic treatment for metastatic disease, as these patients are not naïve and untreated, as was more frequent in the past. The management of patients relapsed at visceral sites after adjuvant treatment represents one of the major challenges in daily clinical practice.

## Figures and Tables

**Table 1:** DMFS at 4 years according to BRAF mutation status.

	Patients Stage	BRAF Mutant			BRAF Wild-Type		
		No.	Active Drug	Comparison Arm	No.	Active Drug	Comparison Arm
COMBI-AD	IIIA ^§^-IIIB-IIIC	870	67%	56%			
KEYNOTE-054 ^§§^	IIIA ^§^-IIIB-IIIC	440	63.7%	43.4%	448	62.1%	51.4%
CHECKMATE 238	IIIB-IIIC-IV	311	59.6%	54.6%	323	55.4%	49.5%

^§^ IIIA only >1 mm metastasis in SLNB; ^§§^ data at 3.5 years.
